# The paediatric change laboratory: optimising postgraduate learning in the outpatient clinic

**DOI:** 10.1186/s12909-016-0563-y

**Published:** 2016-02-02

**Authors:** Mads Skipper, Peter Musaeus, Susanne Backman Nøhr

**Affiliations:** Department for Postgraduate Education, Aalborg University Hospital, Forskningens Hus, Sdr. Skovvej 15, 9000 Aalborg, Denmark; Centre for Health Sciences Education, Aarhus University, Aarhus, Denmark; Department of Clinical Medicine, Aalborg University, Aalborg, Denmark

**Keywords:** Continuing medical education, Change Laboratory, CHAT-cultural historical activity theory, Organisational innovations, Work planning, Hospital, Residency, Outpatient clinic, Paediatrics

## Abstract

**Background:**

This study aimed to analyse and redesign the outpatient clinic in a paediatric department. The study was a joint collaboration with the doctors of the department (paediatric residents and specialists) using the Change Laboratory intervention method as a means to model and implement change in the outpatient clinic. This study was motivated by a perceived failure to integrate the activities of the outpatient clinic, patient care and training of residents. The ultimate goal of the intervention was to create improved care for patients through resident learning and development.

**Methods:**

We combined the Change Laboratory intervention with an already established innovative process for residents, 3-h meetings. The Change Laboratory intervention method consists of a well-defined theory (Cultural-historical activity theory) and concrete actions where participants construct a new theoretical model of the activity, which in this case was paediatric doctors’ workplace learning modelled in order to improve medical social practice. The notion of expansive learning was used during the intervention in conjunction with thematic analysis of data in order to fuel the process of analysis and intervention.

**Results:**

The activity system of the outpatient clinic can meaningfully be analysed in terms of the objects of patient care and training residents. The Change Laboratory sessions resulted in a joint action plan for the outpatient clinic structured around three themes: (1) Before: Preparation, expectations, and introduction; (2) During: Structural context and resources; (3) After: Follow-up and feedback. The participants found the Change Laboratory method to be a successful way of sharing reflections on how to optimise the organisation of work and training with patient care in mind.

**Conclusions:**

The Change Laboratory approach outlined in this study succeeded to change practices and to help medical doctors redesigning their work. Participating doctors must be motivated to uncover inherent contradictions in their medical activity systems of which care and learning are both part. Facilitators must be willing to spend time analysing both historical paediatric practice, current data on practice, and steer clear of organisational issues that might hamper a transformative learning environment. To ensure long-term success, economical and organisational resources, participant buy-in and department leadership support play a major role.

**Electronic supplementary material:**

The online version of this article (doi:10.1186/s12909-016-0563-y) contains supplementary material, which is available to authorized users.

## Background

The healthcare system and doctors’ educational environment form an intertwined social practice in constant change because of change in society, patient demands, medical research, etc. [1, 2]. Research has shown that there are tensions between providing high-quality patient care and postgraduate training [3, 4]. When you make changes in patient care, it has ramifications for postgraduate training and vice versa. Furthermore, each social practice – patient care and postgraduate training – are constantly changing. You cannot step into the same river twice as the classical Greek philosopher Heraclitus purportedly said or as put by Willis:”The behaviour of individuals in an organisation, such as a large public hospital, is forever changing. In the modern organisation, what is predictable and stable is ‘change’; what is unpredictable is its direction” [5]. This quote serves as a reminder that because the cultural and technological practice of a medical department constantly change, medical researchers and practitioners need tools to analyse and intervene in a department’s practice of care and continuing education.

The study was motivated due to a wish to reinforce an already established innovative process, the 3-h meetings [6]. The concept of the 3-h meetings is outlined in Table 1.

**Table 1:** 3-hour meetings

The 3-h meeting, an established practice in hospitals in the northern part of Denmark since 2002, aim to engage residents in generating educational initiatives supported by management [6]. Its key-element is hospital management involving residents in the process of how to improve the educational environment and activities in the clinical work setting. This is done by creating a reflective space and an appreciative inquiry process in each department in the hospital – for 3 h. The meeting comprises reflection, dialogue, and coming up with new ideas. The meeting results in suggestions for action plans, and leads to redesign of training and work, and to implementation of more than hundred educational activities at the hospital each year. Records of the residents’ reflections, action plans, and blue print for action on important educational issues have been collected in an annual electronic report since 2006.	

These meetings aim to improve the educational environment and activities in the clinical work setting and have been a part of practice at the studied university hospital for over 13 years with success. We found a gap in how to handle the continuous challenge of change and organisational development in the realm of the complex organisation of a hospital environment [7]. Therefore, we aimed to contribute to evolve ideas about change and improvement in postgraduate medical education.

Studies have found barriers in the learning environment and teaching of residents [8–10]. In a recent study, Miloslavsky et al. [11] found two domains of barriers and facilitating factors in regard to the resident-fellow teaching interaction: (1) Domain of individual/personal factors such as motivations and perceptions and (2) a domain of systemic factors referring to workflow and workplace culture. Miloslavsky et al. argue that the barriers are amendable for change but there is a need for interventions to reduce these barriers and improve the clinical learning setting [11]. Greenfield et al. [12] advocate the use of action research methods as a way to help change culture and practices in healthcare organisations. However, we might also acknowledge the challenges for insiders and outsiders to affect deep change in something as evasive and encompassing as culture, which is why we turned to the Change Laboratory geared towards whole work activity change.

The Change Laboratory intervention method was developed by Engeström et al. [13] and is a tool to support participants in redesigning their work and the organisation of work. The Change Laboratory aims to result in an expansive learning cycle and to find solutions and construct a new theoretical model for learning activity. The expansive learning process involves questioning and analysing a given activity [14] meaning a social practice such as paediatric work practice. Any human activity is both historically created and created in the present. Thus, medical doctors going about their everyday work create paediatric practice but this work is also the result of a long history of medical technological developments, scientific discoveries, and craftsmanship of paediatricians.

The aim of this article is to spearhead an innovative process in a paediatric department and develop new ways of working and learning at the department. In staying true to the Change Laboratory approach, not only did we want to analyse the organisation of training in a paediatric department, we wanted to reinvent it by modelling it and implementing change in the new activity centred on improved care for patients and learning for residents. In summary, the aim of our study was to employ, develop, and evaluate a modified version of the Change Laboratory approach in conjunction with the established 3-h meetings in a paediatric department at a university hospital in Northern Denmark, based on an increasing need to address the complexity of healthcare organisations activities.

## Methods

### Theoretical framework

**Fig. 1:**
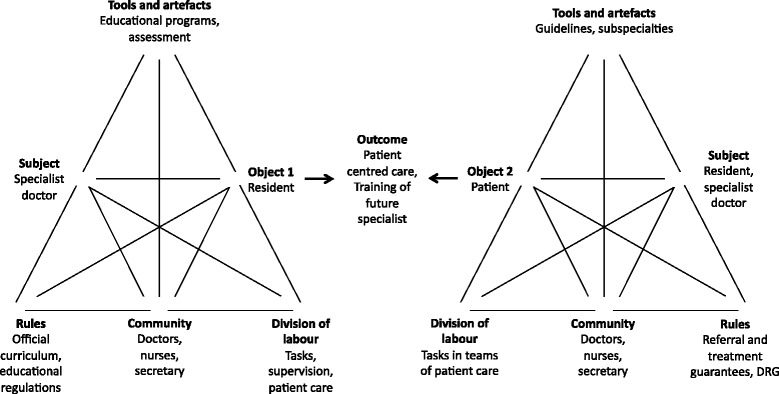
The outpatient clinic as activity systems for residents training and patient care

The theoretical framework behind the Change Laboratory is cultural-historical activity theory abbreviated CHAT. CHAT derives from cultural history through the work of Soviet psychology and people like Vygotsky, Leont’ev, Davydov, Engeström [15] and others. An activity system is conceptually depicted as an entity of human activity in interconnection with six elements: Object, subject, rules, mediating artefacts, community, and division of labour [15] as shown in Fig. 1 with key terms explained in Table 2.

**Table 2:** Definition of key terms in CHAT

(1) The object of the activity: Objects are defined as the meaning or purpose of the activity, which defines and distinguish it from other activities [14].
(2) The activity system: An activity system can act as an object of stimulation in creating change [16].
(3) Contradictions: Contradictions are conceived as part of the multi-voicedness (different perspectives of participants) of an activity, which is the source of tensions, underlying contradictions in the activity. Contradictions are structural tensions between the opposing forces in the activity.
(4) Expansive learning cycle: Contradictions are driving forces of change and they origin from the historically accumulated tensions between activity systems. When the double bind of contradictory demands, made by activity systems, are overcome by participants expansive learning might result.

**Fig. 2:**
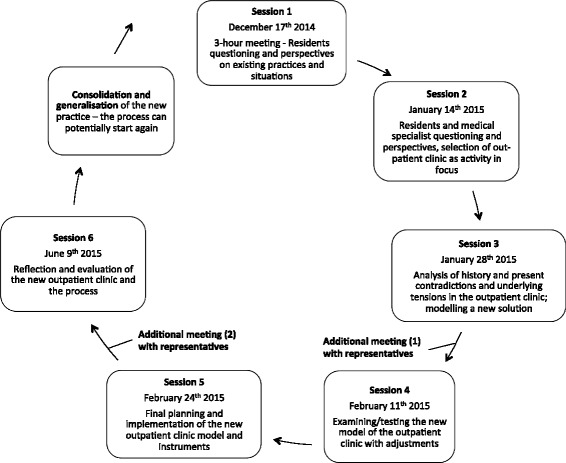
Our sessions as a process of an expansive learning cycle [14]

The crux of CHAT is that the subject, who may be an individual doctor, patient or team, is conceived as part of an activity system working towards an object resulting in an outcome. The subject and object are mediated by signs and tools (material artefacts and ideas) that are used by and which enable the subject to achieve a result by working on the object. This result or outcome is simultaneously influenced by the underpinnings of the activity system: Rules, community / team, and the division of labour. The activity system model is often constituted of two or more activity systems; in our case, we present the outpatient clinic as an activity system for patient care and as an activity system for training residents (Fig. 1). Any change in one of the displayed elements of the activity system can cause changes in the others - the system is essentially unstable. This systemic account conceives of learning as expansive referring to a transformation of the subject (e.g. the individual doctor, a medical team, or an entire system of patient-care provider). CHAT can be used to describe the development (and learning drives development according to CHAT) that can happen in activity systems based on contradictions that participants (doctors) are experiencing. This means that participants’ different experiences and the boundaries and contradictions between the activity systems can be tested and refined during cycles of collaborative inquiry between participants of the activity systems. Thus, the theoretical framework of CHAT helps us to analyse and explore the empirical data of specialist training in the clinical settings during the process of expansive learning. It also helps us to illustrate the complexity of the interacting influences of the system, allowing the participants and us a better understanding of the social and historical factors that influence the learning process. Engeström and colleagues refer to this process of expansive learning as, “another step in developing a learning theory based on ascending from the abstract to the concrete” [17], as shown in Fig. 2.

What is the essence of the Change Laboratory, an intervention, a method, a methodology or a philosophical approach? The Change Laboratory is a social scientific approach involving a cultural historical philosophical analysis and an intervention rather than a methodology with a step-by-step cookbook about actions to take when intending change. We used the Change Laboratory as a reflective tool, which helped us manage organisational change, and in this way it became a method for changing social practice because participants’ redesigned work and learning activities, not just their ideal conceptions of learning and work.

### Context and setting

This intervention study is part of a Ph.D.-research project in a paediatric department of a university hospital in Northern Denmark. Residents work full-time in the department during residency and are a fully integrated part of the daily clinical work routines, responsible for patient care in the paediatric emergency department, in-patient units and outpatient unit under varying levels of supervision and guidance by specialist doctors.

The outpatient unit is composed of four tracks/clinics a day (staffed with 1-2 specialist doctors and 2-3 residents) covering in total nine paediatric sub-specialities. The outpatient unit had approximately 14,000 patient visits in 2014 and an estimated forecast of 18,000 visits in 2015. The department aims for residents to spend in average of one working day a week in the outpatient clinic during residency. For further detail on the educational system of paediatric residency training in Denmark, we refer to Additional file 1.

In total, the department in question comprises of 24 paediatric specialists constituting faculty, and 16 residents. Due to regular replacements among residents during the study, the total number of participating residents was 21.

The Change Laboratory sessions were located in the conference room of the paediatric department, and we used whiteboards and Microsoft PowerPoint presentations as instruments for capturing participants’ ideas and thus promote transformative thinking. We prepared models of activity systems of participants’ learning activities in the paediatric department beforehand and drew them manually on the whiteboard in order to produce signs that could be modified by participants and help participating doctors’ gain a theoretical understanding of their learning environment.

### Mirror-data

The Change Laboratory method employs the concept of Vygotsky’s method of double stimulation where concrete stimuli of daily practices, also referred to as mirror-data, are presented to the participants, but in an ambiguous way leading to a contradiction in terms of what this stimuli means [14]. The mirror-data is chosen not only in order to have multiple meanings, but also to portray historical layers in the department’s artefacts and rules, which govern the activity. We used data from a previous conducted ethnographic field study in three paediatric departments in Denmark, including the department currently investigated [18]. The former study aimed to identify factors that facilitate and impede the organisation of paediatric specialist training and thus provided us with glimpses of daily practices concerning specialist training. Furthermore, we used summaries and minutes from 3-h meetings within the paediatric department from the years 2006-2013, which were analysed and used as historical mirror-data in session 3 and in the research groups’ background analysis (Table 3).

**Table 3:** Historical data from previous 3-hour meetings (2006-2013) concerning training in the outpatient clinic

Planned supervision is not utilised optimally due to:	•Unfamiliarity •Lack of request from trainees •Lack of outreach from trainees •Office work/paper work
Supervision throughout the day is challenged by:	•Missing supervisor •Interruptions •Time - delays
Continuity versus diversity in patient contacts:	•Lack of continuity with own patients •Alignment with other tasks and curriculum

### Data collection

We gathered data from December 2014 to June 2015. A summary of participants and sessions is shown in Table 4. Altogether, we conducted five consecutive sessions and one follow-up session as shown above in Fig. 2.

**Table 4:** Session description

Session Number	Number of doctors participating in total		Length of session (Minutes)
	Residents	Specialists	
1	11	1 (moderator)	180
2	6	9	45
3	8	10	55
4	4	10	55
5	4	8	52
6	5	12	60
Average	6.5	8	55

The boundaries of the Change Laboratory were permeable and not limited to the sessions. In between sessions, the first author met or corresponded by e-mail with representatives from the group of doctors responsible for medical education in the department. In order to involve junior and senior doctors as well as management as participants in the process of discussing preliminary results, we choose meetings and informal e-mail correspondences rather than for instance to conduct interviews, which might lead physicians to become research subjects rather than participants. The assumption was that the Change Laboratory process would uncover contradictions between learning activity systems and healthcare activity systems as constructed as signs by participants and moderator in the sessions. In order to challenge the view of management and participants, and to introduce ambiguous stimuli into the sessions, we conducted a single interview with a consultant that the residents had nominated as being particularly good at supervision and structuring training in the outpatient clinic. The consultant reflected on how the outpatient clinic is organised in regards to learning and the barriers experienced in that regard.

All sessions were videotaped and the main researcher transcribed the recordings. At each session, a co-researcher kept observational and reflectional notes on the session focusing on the process and content of discussions during the sessions. The meetings and the interview conducted between sessions were audio recorded and transcribed verbatim. After each session, key discussions and decisions were documented in a written summary that was distributed to participants. The main researcher wrote self-reflective notes in the process after each session and during the analysis of the research containing notes in a research diary [19].

### Data analysis

Data produced and used for analysis, comprised documents related to session 1 (3-h meeting), video recordings transcripts of each session (except session 1, which were audio-recorded), observational notes conducted by co-researcher during sessions, transcriptions of audio-records and notes from interrelated meetings with representatives of doctors responsible for medical education in the department and one interview with a consultant. Educational programs for the residents were analysed and used as background material in the discussion with the representatives from the group of doctors responsible for medical education.

Transcripts from video and audio recordings were read iteratively and analysed using a data-driven thematic analysis approach to identify major themes [20]. The data was coded by extracting experiences, tensions and solutions presented by the participant in the sessions resulting in an initial set of themes. Furthermore, we used the theoretical framework of CHAT to identify activity system components and contradictions and their relations. The themes were agreed upon between the research-group and further condensed and refined. In the results section we present extract of examples of themes and contradictions found in the analysis.

The Ethical Committee of The North Denmark Region exempted the study from ethical approval by Danish law, i.e. according to the Act on Research Review of Health Research Projects, and the Danish Data Protection Agency approved the study. All participants volunteered to participate and their written informed consent was obtained. All transcripts were anonymous, and each participant was assigned a unique identifier code. No information concerning patients was obtained.

## Results

The results of our modified Change Laboratory intervention encompass the themes, the critical tensions/contradictions, and the proposed solutions and outcome - as well as an evaluation of the process. A summary is given in Table 5 and a further description below. Illustrative excerpts are identified to their source by residents (R), medical specialist (MS) and according to session (S).

**Table 5:** Summary of results: Themes, contradictions and suggestions for solution regarding the outpatient clinic

Themes	Contradictions	Solution
Before		
Introduction	•Records, referrals, patient lists, work-schedule, secretary help, booking system •Lack of introduction •Management decisions	Checklist for introduction period Upgrading introduction
		‘Vision paper’
Preparation	•Lack of time •Expectations •Use of spare time (work-life-balance) •Specialist training in a 37 h work-week	No solution found for extra time for preparation
		‘Vision paper’
Pre-supervision session	•Lack of preparation •Lack of participation •Meeting time – different •Taking time from something else (conference, formal teaching)	Full presence at 8 A.M.
		ALL residents and supervisor participate, EVERY time
During		
Structure	•Subspecialty or individual split •Continuity vs. diversity in patients •Specialist vs. broad skills •Interruption of colleagues •Production versus training •Patient expectation of specialist treatment	Subspecialist structure continuous
		Umbrella outpatient clinic
		Extra time in between supervising specialist’s own patients
		“Open door policy”
Resources	•Increased numbers of clinics •Increased number of patients •Staffing of other functions/tasks (e.g. rounds) •Disengaging consultant for supervision •Illness among staff creates vulnerability •Lack of time – used on documentation and IT	Consultant responsible for medical education as scheduler and work planner
After		
Follow-up on patients	•Brief employments/positions •Availability of specialists/supervisors •Responsibility for continuous patient care •Medical specialist commitment is rewarded with “boomerang”/”rebound” •Increased paperwork •Failure to complete/discharge patients due to lack of decision support	‘Vision paper’ set out expectations of residents
		Subspecialist available for feedback on progress

Training of the future specialist in the outpatient clinic was revealed as one of the key problems. In Fig. 1, the complexity and the different perspectives of training of doctors and treating patients in the outpatient clinic are illustrated as two interacting activity systems.

### Session 1

The first session was based on residents participating in a collective analysis of present educational/training problems in the department by conducting the annual 3-h meeting in the organisation. Possible solutions were raised already in this session, because the residents themselves according to the purpose of the 3-h meetings have to come up with solutions/initiatives for change to present to management [6].

Some of the critical tensions regarding the outpatient clinic revealed in the first session were: Lack of supervisors and residents, formalisation of the scheduled meetings in the morning before seeing patients, time constraints and pressure when awaiting supervision during the day as illustrated by the following citation by one of the residents participating:

*“It is still supervision that is lacking… It differs depending on who is the supervisor, if they [medical specialists] show up at eight o’clock or if they do not. We have also been told many times that when they get down there at eight o’clock, they have nothing to do, because we are not prepared.”* R4, S1

### Session 2

In the second session, we expanded the collective analysis by presenting three results to the group of doctors, including both residents and medical specialists: The residents’ models, mirror-data from session 1, and excerpts of data from previous observations and interviews [20]. This discussion uncovered new aspects of existing in a reality of high demands and thinking about a different future, whereby the participants supplemented and enriched the collective analysis.

“*What favours the outpatient clinic is that the amount of work in the outpatient clinic is increasing. And the future is there; we spend an increasing amount of time in the outpatient clinic compared to inpatient wards. So to optimise it will be very important*.” MS4, S2

As the quote illustrates, at the end of the session the participants agreed upon the focus of the following sessions being on the structure and planning of training in the outpatient clinic.

### Session 3 and 4

In these sessions, the different themes and critical tensions/contradictions revealed by the mirror of empirical data from observations and interviews (summarised in Table 5) were continuously presented and the participants were asked to come up with solutions or suggestions for rearranging the activity. This resulted in an action plan, which was divided into three sections:

#### Before – preparation and expectations

Issues concerned how to ensure presence at planned pre-supervision session in the morning before seeing patients in the outpatient clinic and agreement of the purpose of these sessions.

*“The purpose of supervision must be to benefit the patient and to learn something. It calls for the resident to be prepared and requires they have done some considerations beforehand. If the resident is just awaiting a plan from the supervisor, then it is not supervision.”* MS13, S3

Suggestions for solutions concerned upgrading introduction to the department and making guidelines on how to run the planned pre-supervision session as seen in Table 5.

One of the recurrent contradictions mentioned primarily by the residents, was the benefit of being well prepared on patients and treatment issues, and the problem of securing time for this preparation. Residents in general seemed to agree upon the lack of time for preparation, but some found it necessary and imperative to prepare in their spare time, where others found it unnecessary and conflicting in respect to the collective agreements.

*“I spend around on average of one to one and a half hours of my spare time to prepare the day before an outpatient clinic. It depends on what kind of clinic, but if it is endocrinology or neurology, I do. And then you can argue whether you should do that.”* R17, S4

This quotation from one of the participating residents illustrates a contradiction and a recurring theme to which the group did not find a solution during the sessions – acquisition of time for preparation.

#### During – structural context

The participants held different experiences from other departments on how to best structure and organise the outpatient clinic concerning both continuity in patient care and keeping a subspecialty structure, seeing a diversity of patients. This is illustrated by a medical specialist contending that it is essential for residents to see a diverse spectrum of patients in regard to their training:

“*So you could argue that it is unnecessary to see the same patient four times in a row if it’s for training. If it’s service and if it should benefit the patient, then it may be a worthy argument; but it is not certain that it’s for the best in regards to training.”* MS7, S4

Some of the participants held the view that a restructuring of the outpatient clinic would increase both productivity and the quality of patient care. By allowing several residents to participate in the care of patients, with a medical specialist with the sole function of supervising, the supervising medical specialist would be exposed to all patients seen. However, this would require an increased number of residents in the outpatient clinic, which could bring forth other problems, for example, the staffing of the inpatient wards:

“*Then there is the problem that the residents will be displaced from the inpatient rounds. It is a disadvantage for the inpatient wards. We have to hire more doctors for ends to meet, and we probably won’t get permission to do that.*” R17, S4

Solutions suggested concerned how to restructure the outpatient clinic into a so-called “umbrella”-structure, running parallel patient consultations in separate examination rooms (“Report-back model” [21]), and a “vision paper” to state the guidelines for supervision and the mutual expectations for the group of people around medical training.

#### After – follow up and feedback

Training and developing residents’ professional autonomy and responsibility for patients was a common objective. Several contradictions were found in regards to this, amongst others the fact that some of the residents where only employed/present in the department for six to twelve months, whereas some of the patients were assigned and visiting the outpatient clinic over longer periods, as the following quotation from a medical specialist illustrates:

“*In principle, you are supposed to be responsible for the patient until the next patient visit in the outpatient clinic… I have experienced many times that patient records are being put in my box, because residents have been in a neurology clinic for one day, and then they ask me, ‘what are we going to do with this?’ ‘Would you just look at it?’… And many times, it is from someone who has left [the department] and they cannot follow up on patients*.” MS16, S4

The availability of the specialist for follow-up and feedback was also a challenge due to both different work schedules and an experienced lack of time. The solutions addressed how to provide guidelines to establish procedures and expectations for both residents and medical specialist on how to secure adequate feedback and follow-up on patients.

### Session 5

In the fifth session, the plan for action was presented and discussed in detail and finally accepted by the participants without major revisions. Before the session, a written draft of the action plan was emailed to the entire group of medical doctors in the department even though they did not all participate in the session, which allowed them to comment on the action plan outside the formal session.

Some of the previously mentioned contradictions and tensions were discussed again in light of the suggested plans for action, for an example, the disengaging of consultants for supervision, here illustrated and argued by a medical specialist:

“*The fact is that in my clinic I see many things of which I am certainly overqualified in the light of the fact that I’ve seen the same things the last 20 years, exactly the same type of patients. I do not have to be productive in relation to patients… in the long term it could well be a transition towards senior doctors to supervise more and junior doctors to produce more.*” MS7, S5

The session ended with the appointment of key-persons in the department responsible for the implementation of the different actions.

### Session 6 – follow up

After three months, a sixth and final session was performed where focus was on the status of the action plan and on evaluation of the process. This was in accordance with the dual aim of the process of Change Laboratory, to generate new concepts and solutions resulting in redesigning of the outpatient clinic as a training site, and to establish ownership and agency among participant regarding the intervention process.

In general, the participants agreed that several of the objectives for the suggested solutions and concepts were achieved, amongst others a change in the culture of attendance at the planned pre-supervision sessions furthermore formulating and agreeing on a “vision paper” stipulating the mutual expectations and guidelines for supervision.

“*When the little things are working, it is easier to succeed. The culture has indeed changed, we have had our challenges at getting supervisors to show up and the residents have been waiting, but also the opposite. In my experience, it is the culture that has changed significantly, and hopefully it stays that way*.” MS19, S6

The solution regarding changing the structure of the outpatient clinic into an “umbrella”-structure was not accomplished yet. The head of department, however, stated in the session that he would consider restructuring his own sub-specialty clinics as a pilot to see if it would be feasible, considering the implications it could have on the work planning:

*“It takes some work schedule gymnastics, so it has to be planned in good time, you can’t do it on short notice, but I think it could be a model. It has been done in other departments*.” MS3, S6

The above example illustrates how the Change Laboratory can act as a pilot experiment to develop new solutions and rearranging the activity prospectively [14]. Furthermore, the outcome of the change intervention deepens participants’ and the researchers’ understanding of the underlying nature and complexity of tensions and challenges on how to create change in concepts and solutions benefitting both patient care and training of future specialist doctors.

In the final session, the participants were asked to reflect upon the process of the Change Laboratory and the feedback was positive. The participants felt that the shared reflection on ideas and suggestions for solutions was important, including a shared responsibility for implementation and change, and that the individual medical doctor learned how to influence their own organisation of work. As a resident put it:

“*It takes off some of the grumble over things that don’t work, that you think constructively, what can we do about it… giving the opportunity to influence your own work life*”. R11, S6

The participants agreed that the method could be applicable in other departments, but underlined the importance of local engagement and having an anchor person to guide and manage the process. Suggestions for alterations were to include participants from other professional groups and keeping shorter intervals between the sessions.

## Discussion

In this change intervention study, we used CHAT to conceptualise the paediatric outpatient clinic as two interacting activity systems, the objectives of the activities being training of residents and treating patients (Fig. 1). During intervention, we identified tensions and contradictions in order to make sense of the different practices and learning cultures present in the organisation in focus. This resulted in enhanced mutual understandings and solutions for restructuring the outpatient clinic (Table 5).

We found CHAT useful in order to understand and describe the organisational complexity of a hospital organisation, e.g. an outpatient clinic. This finding is in congruence with Bleakley [22], who highlights CHAT as especially applicable when interested in how learning can cross different teams, e.g. activity systems. The collaborative inquiry of the Change Laboratory method helped us visualise the ‘boundary crossing’ between specialist doctors and residents in understanding the common ‘boundary object’ [22] – patient care - and helped us to study the effect of change in a rapid changing healthcare system within professional practices, as suggested by others [23]. We found the focus on the organisation and the use of historicity very important and useful additions to the 3-h meeting process.

The social tensions delineated in the intervention, as well as participants’ attempts to overcome these tensions by coming up with solutions, are consistent with other studies and recommendations [21, 24–27], and raised a need for solutions aimed at structural and conceptual changes [28]. Although our results may not be directly generalised to other outpatient clinics, other healthcare practitioners and researchers might use the Change Laboratory approach as a way to facilitate change in the organisation of medical education, which forms a complex activity system within any hospital organisation. In this study, it was not our primary aim to come up with general solutions for arranging outpatient clinics as training sites transferrable to any setting in any culture. This would be against the idea of an activity system as bounded by history and culture. Our aim was to illustrate how to use this theoretical framework and method to change practice in paediatric postgraduate education in Denmark.

To improve and carry out the implementation of change, any approach is contingent upon personal interaction and communication, in our case in the community of doctors. The case-based approach allowed the participants to engage in issues relevant for their work environment. We provided the participants with an underlying understanding of their role and position within the organisation, something which is not unique to the Change Laboratory, but in concordance with participatory design studies [29, 30]. Although participants’ novel insights resulted in new approaches towards work based learning, we cannot know for certain if there has been a stable change in the departments’ organisation of learning in the long run. As Virkkunen and Newnham [14] point out, it may only be small outcomes and parts of the process that can be seen by the researcher in the Change Laboratory straightaway because change in an organisation requires that participants employ the new solutions and concepts that they themselves have helped create during the intervention. As succinctly formulated by Virkkunen and Newnham: “For the researcher-interventionists the immediate outcomes of the Change Laboratory process are new insights, ideas and challenging problems of theory and method” [14]. Timing the implementation of the intervention was challenging because it depended on the community’s readiness for participation and consolidation of the solutions and new practices. Working intensively with the health community, in our case with highly trained paediatricians who are part of the medical profession, was only possible after being granted access and the medical profession is known to govern access [2]. The first author, a paediatrician himself, was easily granted initial access by the leader, but had to prove the worth of the intervention study to both residents and specialists (novices and old-timers), session by session, even outside sessions, by prolonged engagement with participants. This supports our point that the Change Laboratory intervention was not a cookbook of ready-made instructions. It was a painstaking, time-consuming but rewarding process that allowed us to create an expansive learning process of both the process itself and the activity in focus: The real and re-imagined outpatient clinic as a training and production site. Creating time and space for managing a change and innovation process within the realm of a busy and complex hospital organisation, was a challenge in itself as shown by others [7]. Factors as political involvement, leadership, resistance from heads of department and low priority of education and change, has been shown to be both inhibiting and conducive factors for implementing curricular change in postgraduate medical education [31]. All though it is beyond the scope of the present study and not further explored, we found that this must be taking into account by others who wish to implement similar change process in their own institutions.

The Change Laboratory method in some parts resembles the process of action research, which is well suited for identifying problems and improving practices in clinical settings [21]. Generalisations made from action research studies differ from those of other more conventional types of research. We aimed to describe the work in as rich detail as possible, and in accordance with guidelines and recommendations on action research and qualitative research [21]. Although participatory action research is not often published in medical education research, it has been used to develop educational materials for general practitioners and facilitating curriculum change in medical schools [32, 33]. Especially we found the integration of the process, in the everyday reality of the activity in focus, essential and useful. This allowed engagement with the participants in the process and allowed us to identify problems and contradictions in the on-going activity and organisation and jointly come up with solutions [14]. The expansive learning cycle provided continuously feedback of findings to the participants during the sessions for further validation and clarification. The researchers’ self-reflective role is highlighted in both action research as well as the Change Laboratory [14, 21], directing us as researchers to visualise and make explicit our own values and impressions as a part of the research process and analysis. Our study differs from action research in some aspects such as data gathering, which was gathered by the researchers and not by the participants themselves. Furthermore, we are reminded of Virkkunen’s point that what separates Change Laboratory methodology from action research is the use of the historical analysis in the process as well the use of the activity system concept and model [34]. Additionally, the Change Laboratory methodology focuses on the entire activity system more than on the individual actions of the participants, in contrary to action research, which tends to focus on the individual’s actions.

Focusing our analysis on the entire activity system, we found that contradictions related to time (for supervision, preparation etc.) were an essential theme. Especially the lack of time and ways to cope with this contradiction was a recurring source of discussion. The challenges and contradictions found concerned amongst others how to prioritise the use of time in regards to both patient care and training. Seen in the perspective of the activity system model (Fig. 1) the division of labour/tasks between the communities of doctors affect this contradiction, but also the rules implemented by government and hospital management e.g. treatment guarantees. The participants sought to resolve some of the problems concerning time by developing guidelines (artefacts) that should address and establish procedures for how residents and medical specialists should go about making room (division of labour) for e.g. feedback and supervision.

The themes and contradictions revealed in our analysis mirror the findings and observations in other studies from elsewhere in Europe and North America [3, 4, 8–11]. An underlying theme of most of these studies as well the present study is the tension between the need for patient care as service and the demand for structure and training of competent specialist doctors. As an example from the present study, this is conveyed by one of the consultants, who advocates for a more coherent view on patient care as service as well as a means for training and underlines the consultants’ role as supervisors for residents. Our findings are also comparable in regard to aforementioned studies in the similarity of positive as well negative factors found stimulating and enabling a supportive learning environment. Especially factors concerning allocation of time, resource limitations and constraints, and support from senior doctors are found to be influential and significant by the group of doctors in our study and in the international arena as well. We do acknowledge that the ratio of doctors per capita can be of substantial difference around the world as well patients’ and society’s expectations of healthcare services. However, the above-mentioned similarities and comparisons would suggest that our results may be generalised to settings outside of Denmark, and that the need for tools and methods to overcome and confront these challenges are ubiquitous.

### Limitations and future research

Our choice of method required and depended upon the acceptance and ownership of the participating community of medical doctors as well as their engagement in the intervention and research process evolving over time. Our participants could have found it difficult to decline participation due to the location (morning conferences) and the peer-pressure of colleagues and consultants responsible for medical education. Although this may have challenged the participants’ ownership to the final solutions and action plan developed, this was not the case - on the contrary, they gave positive feedback on their participation in the final session. The head of the department found the research essential for developing the training opportunities of the department, and thus we had full support from management to conduct the research project and the intervention. This support from management was a contributing factor for successful implementation of the Change Laboratory in the department, perhaps characteristic of the Danish context in which we conducted the intervention. As supported by studies on postgraduate medical education [35, 36], Denmark is considered a country with a low degree of hierarchical power structure. Thus we would expect broad participant acceptance of a change process and access for less powerful novices (residents) of the department to be involved in change process and allowed to indicate conditions that need change without fear of ridicule, reprisal etc. Other countries or cultures face different challenges when adapting a complex learning intervention in a healthcare setting. A suggestion for future research could be to further explore the impact of a specific culture – national culture, medical specialty, ethnicity etc. – as context for the change processes in medical education.

Our decision to intervene during normal work hours and our need for stable participation over time were a challenge. In other words, during a busy workday and week it was not the same medical doctors participating in every session. Furthermore, we would have liked to involve the entire community of the activity system, including nurses and secretary staff, since the Change Laboratory can create a process, which involves all stakeholders and is prone to produce an even more stable outcome. However, due to the limited time of the project and a concern about potential lack of commitment and participation in 5-6 sessions of an hour-long duration in- or outside working hours, we found it more feasible to use the restricted time of a morning conference, limiting the participant group only to include medical doctors. A suggestion for future research would be not only to include other staff-members in the departments, but also the patients’ and families’ perspective in the process. This brings new questions and other subjective and objective positions into the activity system, and may generate new contradictions and tensions concerning the object and outcome of the outpatient clinic visits. We anticipate that this could call forth potential contradictions between patient and trainee relationships, e.g. patient expectations of specialist care, but also accentuate and elucidate the mutual benefits and needs of continuity in care.

## Conclusion

We found that instruments and theoretical models are needed in order to break away from the standard practices in the organisations of health care services and solving challenges within work practices and organisations. Practices and work routines, including the training of future medical specialists, are deeply embedded in the context and the organisation of the work. The interventional and developmental capability of an organisation can be improved by using the expansive learning cycle and the methods outlined in the Change Laboratory interventions and 3-h meetings.

In our setting, we found that the Change Laboratory intervention contributed with solutions and shared responsibility of designing the paediatric outpatient clinic to benefit both patient care and training of residents. The residents had tried to solve this complex problem for several years using the 3-h meeting process, and the amendment of the Change Laboratory intervention made it possible to both build on the residents’ contributions and to involve the senior doctors. To succeed with this process, participating doctors must be motivated to uncover inherent contradictions in their medical activity systems of which care and learning are both part. Facilitators must be willing to spend time analysing both historical medical practice, current data on practice, and organisational issues that might hamper a transformative learning environment. While the approach outlined in this study succeeded to change practices and to help paediatric doctors redesigning their work, economical and organisational resources, participant buy-in and department leadership support play a major role in ensuring long-term success.

## Additional file

Additional file 1:
**Paediatric education in a Danish context [** 37, 38 **].** (DOCX 86 kb)

## References

[1] Rotem A, Bloomfield L, Southon G (1996). The clinical learning environment. Isr J Med Sci.

[2] Cervero RM (2003). Place matters in physician practice and learning. J Contin Educ Health Prof.

[3] Nothnagle M, Anandarajah G, Goldman RE, Reis S (2011). Struggling to be self-directed: residents’ paradoxical beliefs about learning. Acad Med.

[4] Kesselheim JC, Sun P, Woolf AD, London WB, Boyer D (2014). Balancing Education and Service in Graduate Medical Education: Data From Pediatric Trainees and Program Directors. Acad Med.

[5] Willis EM (2010). The problem of time in ethnographic health care research. Qual Health Res.

[6] Ipsen M, Nøhr SB (2009). The three-hour meeting: a socio-cultural approach to engage junior doctors in education. Med Teach.

[7] Kajamaa, A. Collaborative Work Development as a Resource for Innovation and Quality Improvement in Health Care: An Example from a Hospital Surgery. In S. Gurtner & K. Soyez, eds. Challenges and Opportunities in Health Care Management. Springer, 2015 p. 123–134. doi:10.1007/978-3-319-12178-9

[8] Eraut M (2005). Mapping the problems facing the new surgical curriculum. Bulletin of The Royal College of Surgeons of England.

[9] Hendry RG, Kawai GK, Moody WE, Sheppard JE, Smith LCR, Richardson M, et al. Consultant attitudes to undertaking undergraduate teaching duties: perspectives from hospitals serving a large medical school. Med Educ. 2005;39(11):1129–39. 10.1111/j.1365-2929.2005.02320.x 16262809

[10] Kendall ML, Hesketh EA, Macpherson SG (2005). The learning environment for junior doctor training--what hinders, what helps. Med Teach.

[11] Miloslavsky EM, McSparron JI, Richards JB, Puig A, Sullivan AM (2015). Teaching during consultation: factors affecting the resident-fellow teaching interaction. Med Educ.

[12] Greenfield D, Nugus P, Travaglia J, Braithwaite J (2010). Auditing an organization’s interprofessional learning and interprofessional practice: the interprofessional praxis audit framework (IPAF). J Interprof Care.

[13] Engeström Y, Virkkunen J, Helle M, Pihlaja J, Poikela R (1996). The Change Laboratory as a tool for transforming work. Lifelong Learning in Europe.

[14] Virkkunen J, Newnham DS (2013). The change laboratory - a tool for collaborative development of work and education.

[15] Engeström Y (1987). Learning by expanding: an activity-theoretical approach to developmental research.

[16] Kerosuo H, Engeström Y, Kajamaa A (2010). Promoting innovation and learning through change laboratory : an example from Finnish health care. Central European Journal of Public Policy.

[17] Engeström Y, Sannino A, Virkkunen J (2014). On the methodological demands of formative interventions. Mind Cult Act.

[18] Mortensen L, Malling B, Ringsted C, Rubak S (2010). What is the impact of a national postgraduate medical specialist education reform on the daily clinical training 3.5 years after implementation? A questionnaire survey. BMC Med Educ.

[19] Legal info: Executive order on the training of specialists 2007. https://www.retsinformation.dk/Forms/R0710.aspx?id=105100 (in Danish). Accessed 22 Oct 2015.

[20] Skipper M, Nøhr SB, Jacobsen TK, Musaeus P (2015). Organisation of workplace learning: a case study of paediatric residents’ and consultants’ beliefs and practices. Adv Heal Sci Educ.

[21] Meyer J (2000). Using qualitative methods in health related action research. BMJ.

[22] Braun V, Clarke V (2006). Using thematic analysis in psychology. Qual Res Psychol.

[23] Dent JA (2005). AMEE Guide No 26: clinical teaching in ambulatory care settings: making the most of learning opportunities with outpatients. Med Teach.

[24] Bleakley A (2006). Broadening conceptions of learning in medical education: the message from teamworking. Med Educ.

[25] Egan T, Jaye C (2009). Communities of clinical practice: the social organization of clinical learning. Health (London).

[26] Irby D (1995). Teaching and learning in ambulatory care settings: a thematic review of the literature. Acad Med.

[27] Skeff KM, Bowen JL, Irby DM (1997). Protecting time for teaching in the ambulatory care setting. Acad Med.

[28] Schultz KW, Kirby J, Delva D (2004). Medical Students’ and Residents’ preferred site characteristics and preceptor behaviours for learning in the ambulatory setting: a cross-sectional survey. BMC Med Educ.

[29] Bardella IJ, Janosky J, Elnicki DM, Ploof D, Kolarik R (2005). Observed versus reported precepting skills: teaching behaviours in a community ambulatory clerkship. Med Educ.

[30] Keirns C, Bosk C (2008). Perspective: the unintended consequences of training residents in dysfunctional outpatient settings. Acad Med.

[31] Delany C, Watkin D (2009). A study of critical reflection in health professional education: “Learning where others are coming from.”. Adv Heal Sci Educ.

[32] Dewar B, Sharp C (2006). Using evidence: how action learning can support individual and organisational learning through action research. Educ Action Res.

[33] Jippes M, Driessen EW, Majoor GD, Gijselaers WH, Muijtjens AMM, van der Vleuten CPM (2013). Impact of national context and culture on curriculum change: a case study. Med Teach.

[34] Mash B, Meulenberg-Buskens I (2001). “Holding it lightly”: the co-operative inquiry group: a method for developing educational materials. Med Educ.

[35] Mowat H, Mowat D (2001). The value of marginality in a medical school: general practice and curriculum change. Med Educ.

[36] Virkkunen J, Vilela RADG, Querol MAP, Lopes MGR (2014). The change laboratory as a tool for collaborative transforming work activities - interview with Jaakko Virkkunen. Saúde E Sociedade.

[37] Westerman M (2013). The transition to hospital consultant: Denmark and the Netherlands compared on preparedness for practice, perceived intensity and contextual factors. Med Teach.

[38] Jippes M, Majoor GD (2008). Influence of national culture on the adoption of integrated and problem-based curricula in Europe. Med Educ.

