# Supplementary material for: The paediatric change laboratory: optimising postgraduate learning in the outpatient clinic
Source: BMC Med Educ. 2016 Feb 2;16:42. doi: 10.1186/s12909-016-0563-y (PMC4736176; doi:10.1186/s12909-016-0563-y)
Supplement: Additional file 1: — Paediatric education in a Danish context [ 37, 38 ]. (DOCX 86 kb) [file 12909_2016_563_MOESM1_ESM.docx]

| Appendix 1: *Paediatric education in a Danish context* |
| --- |
| In Denmark, undergraduate medical education lasts six years. After graduation, a mandatory year of foundation/internship training precedes specialist training and followed by an introduction-year post in a preferred speciality. Trainees can undertake up to four introduction-year posts before applying through regional postgraduate training secretaries for specialist training. Specialist training varies from four to five years depending on the speciality and takes place in public hospitals administrated and owned by the Danish Regions – regional governments.  Paediatric specialist training consist of the aforementioned introduction-year post followed by four years of training in residency post divided into two years of training at each of two paediatric departments, respectively a university hospital with specialist functions and a general regional hospital with more general paediatric functions. After completion of specialist training, the paediatric specialist can further specialise in paediatric sub disciplines like e.g. paediatric gastroenterology.  The Danish specialist training is a competency- and time-based medical education, mainly supported by the competency framework like CanMEDs originating from Canada [37]. In Denmark, the Danish Health Authorities (DHA) issue rules and regulations regarding specialist training and hence regulate residency-training [38]. The scientific medical societies outline the formal curriculums of specialist training and the DHA finally approves them. |
